# Supplementary figures and images for: Solitary phytoplankton cells sink in the mesopelagic ocean
Source: PLoS One. 2025 Jul 8;20(7):e0321918. doi: 10.1371/journal.pone.0321918 (PMC12237049; doi:10.1371/journal.pone.0321918)

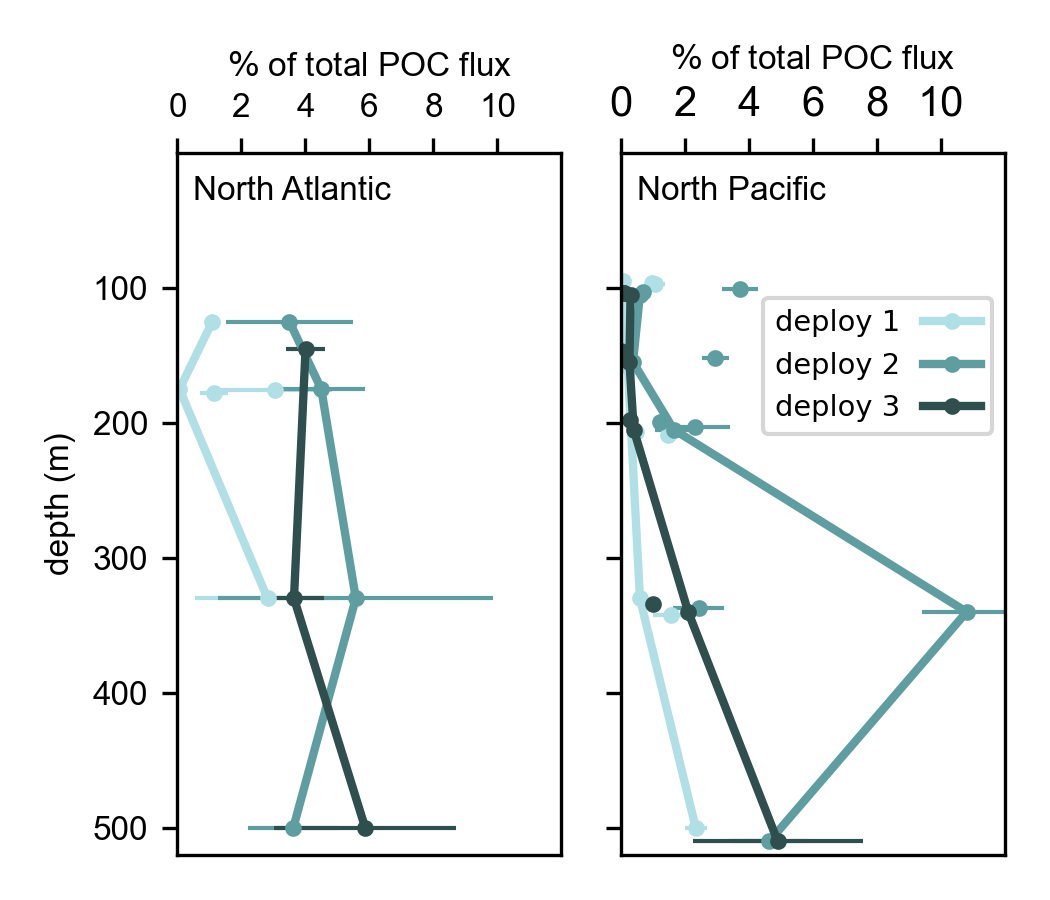

Supplement: S1 Fig — Lines connect samples from the same surface tethered sediment trap (STT) array during each deployment period. Unconnected circles are fluxes measured in neutrally buoyant sediment traps (NBSTs) deployed individually at similar depth as the STTs during each deployment. POC fluxes by solitary cells were calculated assuming an average diameter of 45 µm and using the diatom-specific equations (50). Uncertainties represent the propagated uncertainty of both the cell counting uncertainty and the POC measurement uncertainty. (TIFF) [file pone.0321918.s001.tiff]

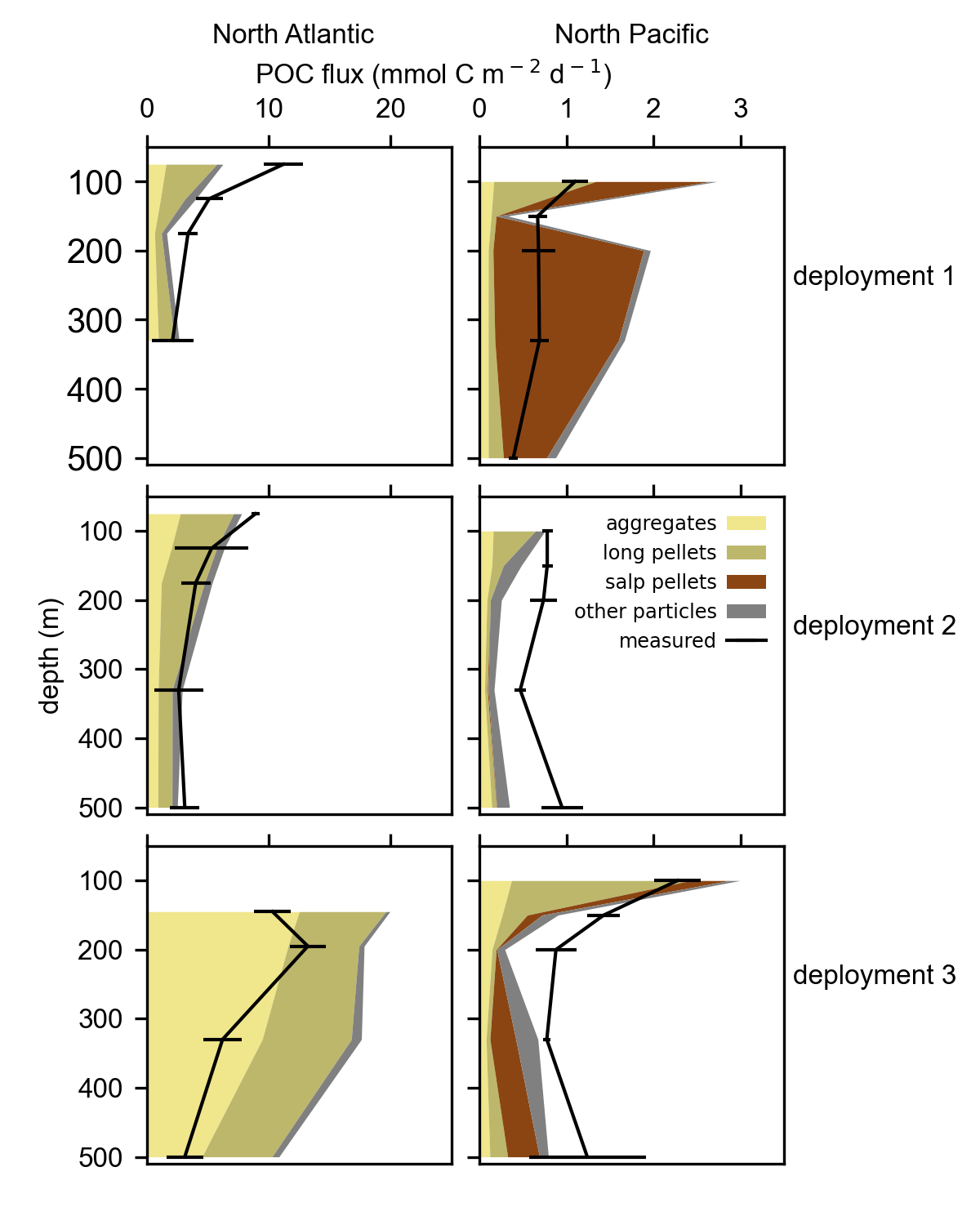

Supplement: S2 Fig — Chemical measurements of bulk POC flux are shown to ground truth fluxes calculated for specific particle types based on imagery. Uncertainties in the measured POC fluxes represent the standard deviation of replicate subsamples. (TIFF) [file pone.0321918.s002.tiff]

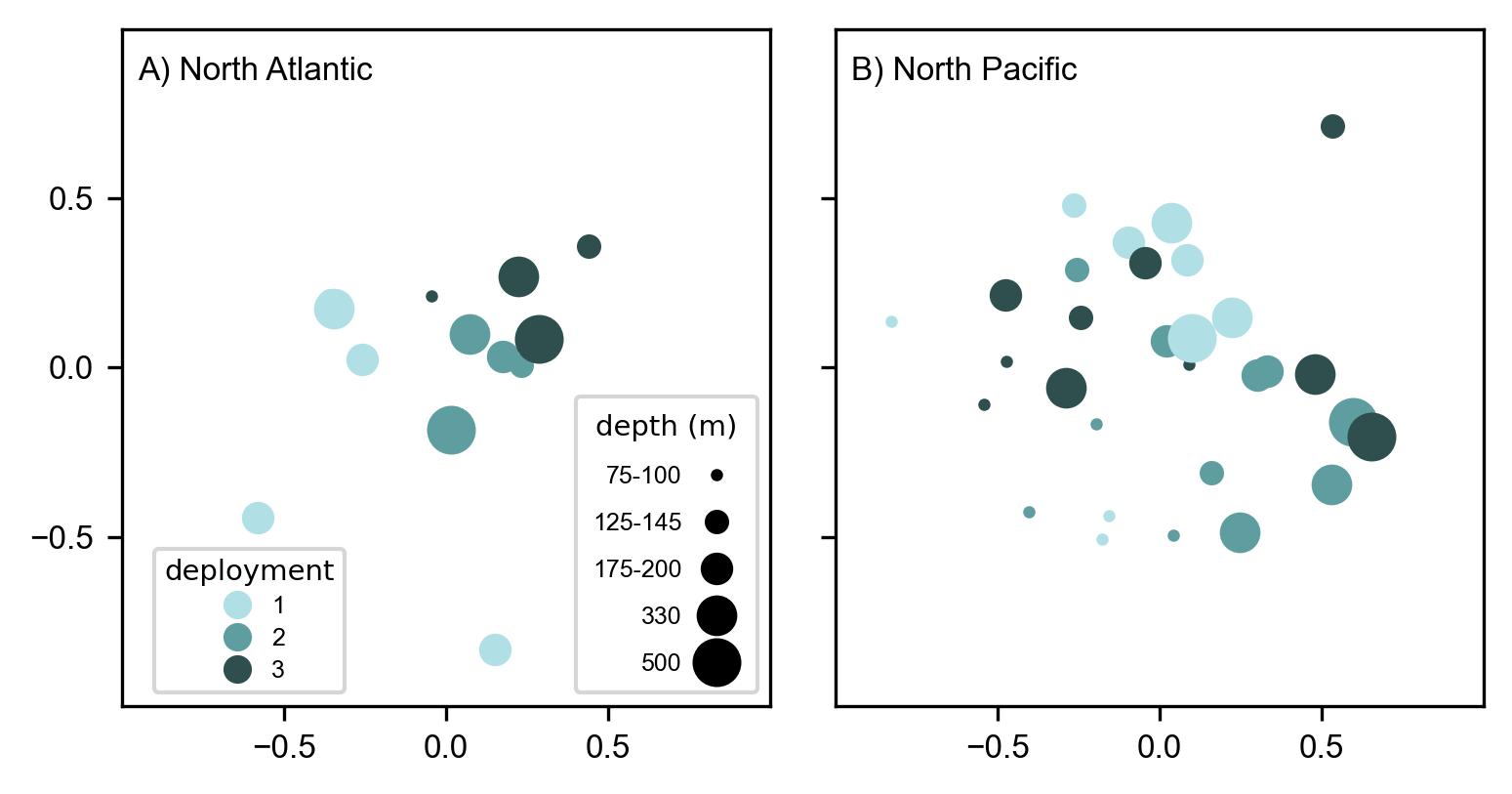

Supplement: S3 Fig — A) North Atlantic Ocean and B) North Pacific Ocean. Distance between samples (dots) on the Multidimensional Scaling plot represents dissimilarity among sample communities. Deployment periods are represented by color and depths are represented by dot size. Phytoplankton sinking in the North Atlantic differed most over time (deployment 1 vs. 2 & 3) while phytoplankton sinking in the North Pacific differed most across depth (95 m versus all other depths). (TIFF) [file pone.0321918.s003.tiff]

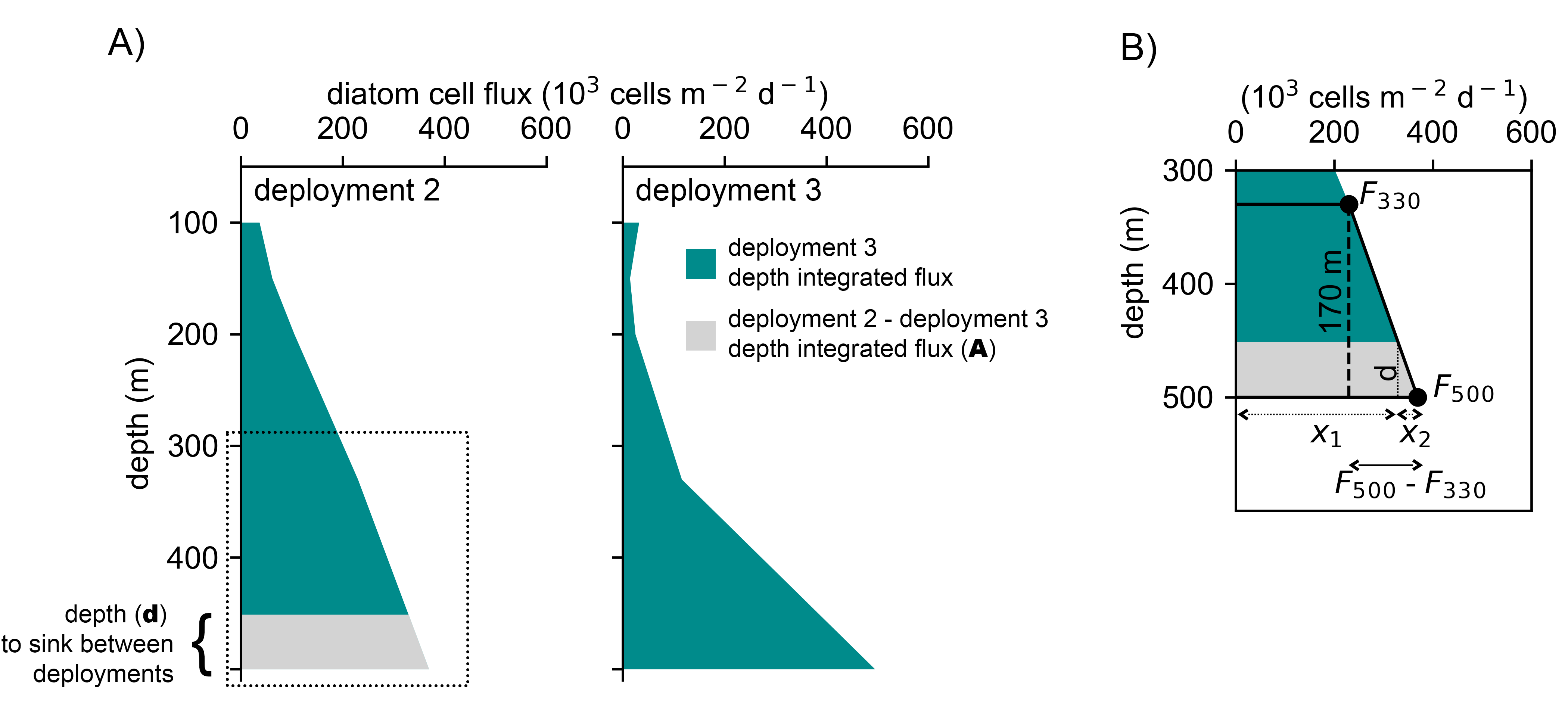

Supplement: S4 Fig — A) The difference in depth-integrated cell fluxes between deployments 2 and 3 in the North Pacific. The gray integrated flux area illustrates the amount of depth-integrated flux reduced during deployment 3. Dashed box is shown in panel B. B) Known and unknown variables of the integrated depth flux profile used to calculate the depth (d) over which cells would have to sink in the 7 days between trap deployments. (TIF) [file pone.0321918.s004.tif]
