# Supplementary material for: Solitary phytoplankton cells sink in the mesopelagic ocean
Source: PLoS One. 2025 Jul 8;20(7):e0321918. doi: 10.1371/journal.pone.0321918 (PMC12237049; doi:10.1371/journal.pone.0321918)
Supplement: S1 Table — (DOCX) [file pone.0321918.s005.docx]

**Supplementary Information**

**Calculation of *Fragilariopsis* sinking speeds**

The depth-integrated loss term (A) for gravitationally settling *Fragilariopsis* diatom cells in the North Pacific (deployment 3 minus deployment 2 depth-averaged fluxes) was used to calculate the depth interval (d) above 500 m during deployment 2 that cells would have to sink in the 7 days before deployment 3 (Supplementary Fig. 4).

The slope (m) of increasing fluxes between 330 m (F_330_) and 500 m (F_500_)

$m=\frac{170 m}{F_{500}-F_{300}}=0.002 m$^3^ d cell^-1^

can also be used to calculate the depth interval (d),

$$d=\mathrm{mx}_{2}$$

and rearranged to

$$x_{2}=\frac{d}{m}$$

where x_2_ is one of two unknown addends of the total flux at 500 m.

F_500_ = x_1_ + x_2_

and rearranged to

$$x_{1}=F_{500}-x_{2}= F_{500}- \frac{d}{m}$$

The known loss (A) of sinking cells during deployment 3 can also be represented by the area of a trapezoid at the base of the deployment 2 flux profile,

$$A=dx_{1}+ 0.5dx_{2}$$

And variable reduced to using equations for x1 and x2:

$$A= {d(F}_{500}- \frac{d}{m})+0.5d \frac{d}{m}$$

which simplifies to:

$$A= F_{500}d- \frac{d^{2}}{m}+ \frac{0.5d^{2}}{m}$$

$$A= F_{500}d- \frac{0.5d^{2}}{m}$$

$$0= \frac{0.5d^{2}}{m}+F_{500}d-A$$

from which d can be solved for using the quadratic equation. Here, d was equal to 46 m, which, when divided by the 7 days between deployment, equates to a sinking speed of 6.6 m d^-1^.

**Supplementary Table**

**S1 Table. Summary of all sediment trap deployments in the North Atlantic and North Pacific study sites, including location, deployment days, trap platform type, depths of deployments in meters, and deployment duration in days.**

| Location | Dates | Platform | Depths (meters) | Collection  duration (days) |
| --- | --- | --- | --- | --- |
| North Atlantic 49°N, 14.9°W | 5 May - 11 May 2021  deployment 1 | NBST | 177, 178 | 1.74–1.92 |
|  |  | STT | 75,125,175,330 | 2.3–5.9 |
| North Atlantic  49.1°N, 14.8°W | 14 May - 16 May 2021  deployment 2 | STT | 75, 125, 175, 330, 500 | 2.78 |
|  |  |  |  |  |
| North Atlantic  49.9°N, 14.8°W | 23 May - 25 May 2021  deployment 3 | NBST | 109 | 1.63 |
|  |  | STT | 145, 195, 330, 500 | 1.82 |
| North Pacific  50.1°N, 145.1°W | 15 Aug - 21 Aug 2018  deployment 1 | NBST | 96, 97, 159, 207, 209, 342 | 4.69–5.27 |
|  |  | STT | 95, 330, 500 | 5.65 |
| North Pacific  50.4°N, 145.1°W | 24 Aug - 28 Aug 2018  deployment 2 | NBST | 101, 103, 152, 199, 203, 337 | 2.80–4.08 |
|  |  | STT | 105, 155, 205, 340, 510 | 4.34 |
| North Pacific  50.6°N, 144.9°W | 31 Aug - 5 Sept 2018  deployment 3 | NBST | 104, 104, 147, 198, 334 | 3.92–4.98 |
|  |  | STT | 105, 155, 205, 340, 510 | 5.33 |
